# Costs of Assisted Home Dialysis: A Single-Payer Canadian Model From Manitoba

**DOI:** 10.1016/j.xkme.2021.04.019

**Published:** 2021-07-07

**Authors:** Ryan J. Bamforth, Alain Beaudry, Thomas W. Ferguson, Claudio Rigatto, Navdeep Tangri, Clara Bohm, Paul Komenda

**Affiliations:** 1 Department of Community Health Sciences, University of Manitoba, Winnipeg, Manitoba, Canada; 2 Chronic Disease Innovation Centre, Winnipeg, Manitoba, Canada; 3 Department of Medicine, University of Manitoba, Winnipeg, Manitoba, Canada

**Keywords:** Dialysis, peritoneal dialysis, hemodialysis, cost, assisted, economics

## Abstract

**Rationale & Objective:**

The prevalence of kidney failure is increasing globally. Most of these patients will require life-sustaining dialysis at a substantial cost to the health care system. Assisted peritoneal dialysis (PD) and assisted home hemodialysis (HD) are potential alternatives to in-center HD and have demonstrated equivalent outcomes with respect to mortality and morbidity. We aim to describe the costs associated with assisted continuous cycling PD (CCPD) and assisted home HD.

**Study Design:**

Cost minimization model.

**Setting & Population:**

Adult incident maintenance dialysis patients in Manitoba, Canada.

**Intervention:**

Full- and partial-assist home HD and CCPD. Full-assist modalities were defined as nurse-assisted dialysis setup and takedown performed by a health care aide, whereas partial-assist modalities only included nurse-assisted setup. Additionally, full-assist home HD was evaluated under a complete care scenario with the inclusion of a health care aide remaining with the patient throughout the duration of treatment.

**Outcomes:**

Annual per-patient maintenance and training costs related to assisted and self-care home HD and CCPD, presented in 2019 Canadian dollars.

**Model, Perspective, & Time Frame:**

This model took the perspective of the Canadian public health payer using a 1-year time frame.

**Results:**

Annual total per-patient maintenance (and training) costs by modality were the following: full-assist CCPD, $75.717 (initial training costs, $301); partial-assist CCPD, $67,765 ($4,385); full-assist home HD, $47,862 ($301); partial-assist home HD, $44,650 ($14,813); and full-assist home HD (complete care), $64,659 ($301).

**Limitations:**

This model did not account for costs taken from the societal perspective or costs related to PD failure and modality switching. Additionally, this analysis reflects only costs experienced by a single center.

**Conclusions:**

Assisted home-based dialysis modalities are viable treatment options for patients from a cost perspective. Future studies to consider graduation rates to full self-care from assisted dialysis and the cost implications of respite care are needed.

## Graphical abstract

**Figure:**
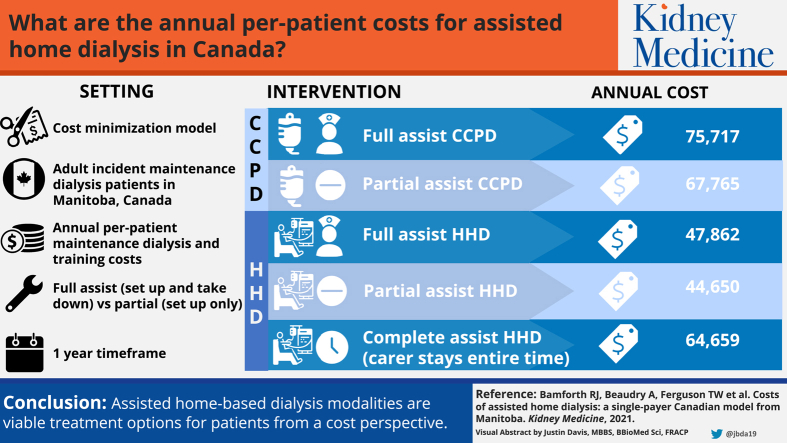

**Plain-Language Summary**

Providing dialysis care to patients with kidney failure is costly for the health system. Performing dialysis in the patient’s home has been shown to cost less in comparison to care provided in the hospital, yet many patients are unable to perform the self-care required. In this study, we provide the yearly cost of providing dialysis in the patient’s home with the assistance of trained caregivers on both a partial and fully assisted basis. Costs such as staffing, dialysis supplies, dialysis machines, utilities, and training were included. The cost model showed that both partial and fully assisted dialysis care offer cost savings when compared with dialysis care provided in the hospital.

Transplantation is the optimal treatment for kidney failure from both quality-of-life and cost perspectives.^1^, ^2^, ^3^ Although the volume of kidney transplants has been increasing,^4^ the demand for donor organs continues to exceed the supply. As such, most patients with end-stage kidney disease (ESKD) rely on life-saving dialysis therapies as treatment for their ESKD.

The provision of dialysis care for patients with ESKD imposes high cost burdens on health care systems due to its resource-intensive nature. In the United States, yearly health care spending on dialysis patients is on average $90.6 thousand per patient.^5^ Overall, Medicare expenditures related to the provision of care for patients with ESKD totaled more than US $35.9 billion in 2017, translating to ~7.2% of total Medicare paid claims.^6^ Most dialysis therapy is provided in the form of in-center hemodialysis (HD), despite evidence suggesting that home-based therapies may offer cost savings in comparison.^7^, ^8^, ^9^ Certain patients may be unable or unwilling to perform full home-based self-care due to cognitive and physical barriers, as well as lack of familial or community support.^10^, ^11^, ^12^, ^13^ Alternative forms of assisted dialysis delivery should be considered to accommodate this patient population, which may offer decreased costs and improved quality of life.

Assisted home peritoneal dialysis (PD) and assisted home HD are alternative care options suitable for patients who are unable or unwilling to perform self-care in home. Assisted PD programs in European countries such as France and Belgium are already well established, demonstrating acceptable quality-of-life and equivalent clinical outcomes in terms of peritonitis, technique survival, and hospital readmissions.^14^, ^15^, ^16^, ^17^, ^18^ Assisted programs implemented in Canadian provinces such as Ontario and more recently British Columbia have demonstrated similar results.^19^^,^^20^ Moreover, in comparison to self-care PD, assisted-PD patients have demonstrated lower transfer rates to HD.^18^^,^^21^ Thus, expansion of assisted-PD programs may mitigate the negative economic and clinical implications associated with modality transfer.^20^^,^^22^ In contrast, assisted home HD programs are less widespread. One reason for this may stem from the lack of uptake of self-care home HD in comparison to competing modalities.^23^ Notwithstanding this, studies evaluating small assisted home HD programs for patients with both mental and physical comorbid conditions suggest that it is both safe and cost-effective in comparison to in-center HD.^24^^,^^25^

The purpose of our study is to provide a descriptive yearly cost analysis of assisted home-based dialysis modalities for patients with kidney failure at varying levels of assistance.

## Methods

Following the methods outlined in Beaudry et al^26^ (2018), this cost-minimization model is presented from the perspective of the Canadian single-payer health system and includes all direct and indirect dialysis-related costs such as direct labor, supplies, equipment, in-center utilities, dialysis-related drugs, overhead, training, and capital costs.^26^ Inputs related to employee benefits and utility costs have been updated from the previous cost model to reflect current rates,^27^, ^28^, ^29^ and the analysis of PD now accounts for cost differences between continuous ambulatory (CAPD) and continuous cycling PD (CCPD).

Furthermore, we build on the original model by incorporating home assistance–specific human resource expenditures related to transportation, parking, and cellular telephones to project the costs borne over time associated with full and partial nurse-assisted CCPD and thrice-weekly conventional home HD. Full-assist CCPD and home HD include a licensed practical nurse performing patient setup, with takedown duties performed by a health care aide. Partial-assist CCPD and home HD include nurse-assisted setup only. Additionally, full-assist home HD was evaluated with the inclusion of a health care aide remaining with the patient throughout the duration of treatment (4 hours) along with performing patient takedown (complete-assist scenario). Scenario descriptions can be found in Table 1.

**Table 1:** Scenario Description

Therapy	Description
Full-assisted CCPD (daily)	Licensed practical nurse performs patient setup, takedown performed by health care aide
Partial-assisted CCPD (daily)	Licensed practical nurse performs patient setup only
Full-assisted home HD (3×/wk)	Licensed practical nurse performs patient setup, takedown performed by health care aide
Partial-assisted home HD (3×/wk)	Licensed practical nurse performs patient setup only
Assisted home HD complete care (3×/wk)	Licensed practical nurse performs patient setup, health care aide remains with patient throughout treatment (4 h) and performs takedown

Abbreviations: CCPD, continuous cycling peritoneal dialysis, HD, hemodialysis.

Modality-specific training costs are incorporated explicitly to understand the impact of this up-front investment on total cost minimization. CCPD and CAPD training times were averaged between modalities to account for potential patient graduation from CCPD to CAPD. Added human resource–related expenditures and model inputs were sourced from the Manitoba Renal Program’s administrative data and in consultation with nurse managers in the Seven Oaks General Hospital dialysis program.^27^ Due to the limited time horizon considered in this analysis, costs were reported without applying discount rates. Study approval from a research ethics board was not sought because only previously published estimates, publicly available information, and aggregate cost estimates were used.

The primary outcome considered was the 1-year maintenance cost of providing dialysis care in Manitoba. Secondary outcomes included training costs encompassing all costs related to direct human resources, benefits, relief hours, and vacation time, as well as the time to reach cost neutrality when comparing modalities. Cost neutrality points represent the points in time at which the most cost-effective modality changes. Moreover, when modalities require an investment in patient training, the model provides estimates for the time-to-payoff–associated costs, given possible alternative modalities.

As sensitivity analysis, in-center HD yearly maintenance costs were varied by ±25% to determine new cost-neutrality points between modalities. Scenario analyses were performed by increasing the dialysis care schedule for home HD patients from 3 to 3.5 times per week. Additionally, we examined the per-patient annual cost by modality in a model in which all assisted home dialysis tasks performed by a licensed practical nurse were instead done by a health care aide. Outcomes are presented in real 2019 Canadian dollars on a per-patient per-year basis.

## Results

**Figure 1:**
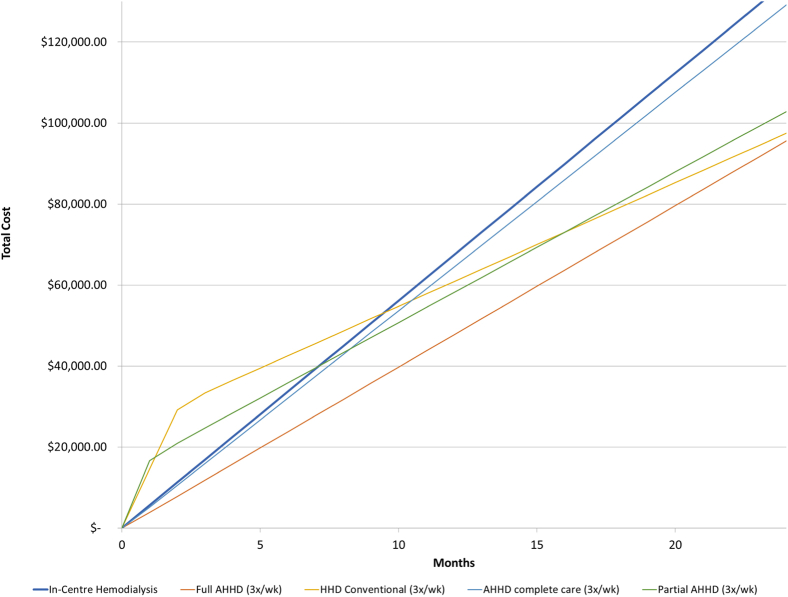
Net accumulated costs over time: assisted home hemodialysis (AHHD) and in-center HD.

**Figure 2:**
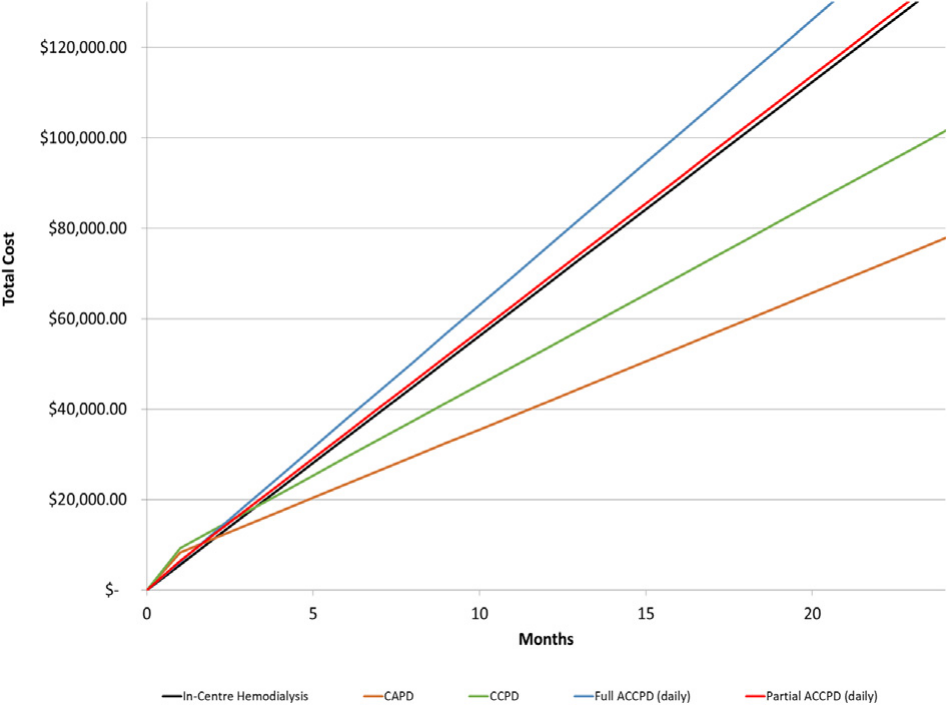
Net accumulated costs over time: continuous cycling assisted peritoneal dialysis (ACCPD) and in-center hemodialysis. Abbreviation: CAPD, continuous ambulatory peritoneal dialysis.

Model outputs overlaid in Fig 1 include net accumulated total costs over time for home HD modalities and in-center HD. PD modality and in-center HD net accumulated costs over time are found in Fig 2. Annual maintenance and initial training costs by modality are included in Tables 2 and 3, respectively, with costs inclusive of maintenance and training at 3, 6, and 12 months outlined in Table 4. In-center HD annual maintenance costs were re-estimated from the previous cost model to reflect changes in benefits and utility costs and totaled $67,416 with no associated training costs.^26^

**Table 2:** Annual Per-Patient Cost of Dialysis Maintenance Therapy by Modality in Manitoba, Canada (2019 Canadian Dollars)

	In-Center HD	PD (CAPD)	PD (CCPD)	Home HD Conventional (3×/wk)	Full-Assisted Home HD (3×/wk)	Partial-Assisted Home HD (3×/wk)	Assisted Home HD Complete Care (3×/wk)	Partial-Assisted CCPD (daily)	Full-Assisted CCPD (daily)
Human resources (direct)									
Registered nurse	$19,594.00	$1,933.44	$1,933.44	$1,109.94	$1,342.67	$1,342.67	$1,342.67	$3,132.89	$3,132.89
Unit clerk	$1,163.65	$295.39	$295.39	$268.53	$268.53	$268.53	$268.53	$295.39	$295.39
Licensed practical nurse	$9,608.66	—	—	—	$5,267.91	$5,267.91	$5,267.91	$12,291.78	$12,291.78
Renal dietician	$685.35	$472.65	$472.65	$567.19	$567.19	$567.19	$567.19	$472.65	$472.65
Dialysis technician	$645.17	—	—	$1,943.01	$1,943.01	$1,943.01	$1,943.01	—	—
Clinical pharmacist	$428.79	$327.90	$327.90	$870.19	$870.19	$870.19	$870.19	$327.90	$327.90
Social worker	$469.20	$383.18	$383.18	$383.18	$383.18	$383.18	$383.18	$383.18	$383.18
Health care aide	—	—	—	—	$2,295.05	—	$14,321.48	—	$5,280.04
Total human resources	$32,594.81	$3,412.56	$3,412.56	$5,142.03	$12,937.71	$10,642.66	$24,964.15	$16,903.79	$22,183.83
Benefits	$6,567.85	$687.63	$687.63	$1,036.12	$2,606.95	$2,144.50	$5,030.28	$3,406.11	$4,470.04
Vacation and relief	$6,395.10	$669.54	$669.54	$1,008.87	$2,538.38	$2,088.09	$4,897.97	$3,316.52	$4,352.47
Milage, communications, etc	—	—	—	—	$330.57	$330.57	$330.57	$745.24	$1,304.16
Supplies: medical, surgical, and laboratory	$7,844.36	$24,487.64	$36,295.34	$10,399.47	$10,399.47	$10,399.47	$10,399.47	$36,295.34	$36,295.34
Supplies: other (eg, housekeeping, maintenance)	$837.82	$333.64	$333.64	$2,907.54	$2,907.54	$2,907.54	$2,907.54	$333.64	$333.64
Drug expenses	$6,004.55	$3,236.88	$3,236.88	$3,184.98	$3,184.98	$3,184.98	$3,184.98	$3,236.88	$3,236.88
Equipment expenses	$580.35	—	—	$4,528.04	$4,528.04	$4,528.04	$4,528.04	—	—
Departmental sundry/miscellaneous	$141.15	$147.08	$206.12	$115.33	$132.48	$127.92	$156.39	$236.67	$249.96
Hospital utilities/overhead (electricity/heat)	$222.40	$77.76	$77.76	$77.76	$77.76	$77.76	$77.76	$77.76	$77.76
Water	$428.17	—	—	$403.67	$403.67	$403.67	$403.67	—	—
Capital cost	$5,798.99	$3,212.65	$3,212.65	$3,212.65	$3,212.65	$3,212.65	$3,212.65	$3,212.65	$3,212.65
In-center runs	—	—	—	$4,601.92	$4,601.92	$4,601.92	$4,601.92	—	—
Total	$67,415.55	$36,265.39	$48,132.13	$36,618.37	$47,862.12	$44,649.77	$64,695.38	$67,764.61	$75,716.74

Abbreviations: CAPD, continuous ambulatory peritoneal dialysis; CCPD, continuous cycling peritoneal dialysis; HD, hemodialysis; PD, peritoneal dialysis.

**Table 3:** Training Cost Overview by Modality in Manitoba, Canada (2019 Canadian dollars)

	In-Center HD	PD (CAPD)	PD (CCPD)	Home HD Conventional (3×/wk)	Full-Assisted Home HD (3×/wk)	Partial-Assisted Home HD (3×/wk)	Assisted Home HD Complete Care (3×/wk)	Partial-Assisted CCPD (daily)	Full-Assisted CCPD (daily)
Human resources (direct)									
Registered nurse	—	$4,806.75	$4,806.75	$15,225.85	$215.17	$7,612.93	$215.17	$2,403.38	$215.17
Clerk	—	$250.63	$250.63	$44.76	—	$22.38	—	$125.32	—
Renal technician/licensed practical nurse	—	—	—	—	—	—	—	—	—
Dietician	—	$223.78	$223.78	$179.02	—	$179.02	—	$223.78	—
Dialysis technician	—	—	—	$2,398.90	—	$2,398.90	—	—	—
Clinical pharmacist	—	$71.61	$71.61	$71.61	—	$71.61	—	$71.61	—
Social worker	—	$313.29	$313.29	$313.29	—	$313.29	—	$313.29	—
Total human resources	—	$5,666.06	$5,666.06	$18,233.43	$215.17	$10,598.13	$215.17	$3,137.37	$215.17
Benefits	—	$1,141.71	$1,141.71	$3,674.04	$43.36	$2,135.52	$43.36	$632.18	$43.36
Relief hours and vacation	—	$1,111.68	$1,111.68	$3,577.40	$42.22	$2,079.35	$42.22	$615.55	$42.22
Total	—	$7,919.45	$7,919.45	$25,484.87	$300.74	$14,813.00	$300.74	$4,385.10	$300.74

Abbreviations: CAPD, continuous ambulatory peritoneal dialysis; CCPD, continuous cycling peritoneal dialysis; HD, hemodialysis; PD, peritoneal dialysis.

**Table 4:** Net Accumulated Costs at 3, 6, and 12 Months by Modality in Manitoba, Canada (2019 Canadian dollars)

Months	In-Center HD	Home HD Conventional (3×/wk)	Full-Assisted Home HD (3×/wk)	Partial-Assisted Home HD (3×/wk)	Assisted Home HD Complete Care (3×/wk)	PD (CAPD)	PD (CCPD)	Full-Assisted CCPD (daily)	Partial-Assisted CCPD (daily)
3	$16,853.89	$33,381.73	$11,811.76	$24,673.86	$15,907.83	$14,331.73	$17,298.41	$18,851.53	$17,786.83
6	$33,707.78	$42,536.32	$23,777.29	$35,836.30	$32,081.68	$23,398.07	$29,331.44	$37,780.72	$34,727.99
12	$67,415.55	$60,845.51	$47,708.35	$58,161.18	$64,429.37	$41,530.77	$53,397.51	$75,639.09	$68,610.29

Abbreviations: CAPD, continuous ambulatory peritoneal dialysis; CCPD, continuous cycling peritoneal dialysis; HD, hemodialysis; PD, peritoneal dialysis.

The annual maintenance cost of delivering daily full-assist CCPD inclusive of direct labor, consumables, and capital totalled $75,717 per patient, with initial training costs of $301. When considering partial-assist CCPD, the yearly per-patient maintenance costs are reduced to $67,765, with initial training costs of $4,385. In comparison, the annual maintenance costs of self-administered CCPD totaled $48,132, with associated training costs of $7,919. The marked cost premium for assisted CCPD over standard self-administered PD is attributed to a significantly higher labor requirement (inclusive of benefits and vacation and relief), with premiums of $36,387 (excluding training costs per year, respectively, vs the self-administered counterpart). This premium is reduced to $19,632 when considering partial-assist CCPD. The annual maintenance and initial training costs associated with self-care CAPD amounted to $36,265 and $7,919 per patient, respectively.

Annual maintenance cost per patient associated with full-assist thrice-weekly home HD using a conventional HD machine was estimated at $47,862, with associated training costs of $301. Maintenance costs for partial-assist home HD were $44,650 per patient per year, with an initial training cost of $14,813. Considering the complete-care scenario in which the health care aide remains throughout the duration of treatment, the annual maintenance cost per patient was $64,695, with training costs of $301. Self-care home HD annual maintenance and initial training costs were re-estimated from the previous cost model and totaled $36,618 and $25,486 per patient, respectively.^26^ Full- and partial-assist home HD are less costly than self-administered home HD in the first year inclusive of training costs, and all assisted home HD scenarios considered in this analysis are less costly per year in comparison to in-center HD. The most cost-effective modality is suggested based on a patient’s projected time receiving a given form of dialysis.

Considering graduation from assisted to self-care dialysis, this model estimates a cost-savings of $27,585 per year when patients graduate from full-assist CCPD to its self-care alternative (excluding training costs). These cost savings are reduced to $19,632 per year when considering graduation from partial-assist CCPD to self-care CCPD. Inclusive of training costs, economic gains are still realized within the first year. Graduation from full, partial, and complete care assisted home HD modalities to self-care home HD translates to a cost savings of $11,244, $8,031, and $28,077 per patient per year (excluding training costs). Inclusive of training costs, graduating from complete care assisted home HD to its self-care alternative is the only scenario in which cost savings are realized within the first year.

Cost-neutrality points representing the time in which cost savings are realized between modalities are presented in Table 5. Between in-center HD and self-care CAPD, cost savings are achieved at 2.94 months. Self-care CCPD was found to be less costly than in-center HD at 4.76 months, with its partial- and full-assist alternatives always being more costly. Considering self-care home HD, cost savings are achieved at 9.44 months compared with in-center HD. The cost-neutrality point between full-assist HD and in-center HD was calculated at 0.09 month. Between partial-assist home HD and in-center HD, cost-savings were achieved at 7.12 months. The point at which cost savings were realized between complete care assisted home HD treatment and in-center HD was calculated at 0.02 month.

**Table 5:** Months of Therapy Required to Achieve Cost Savings

Evaluated Therapy	Comparator Therapy	Months Required for Cost Savings	Sensitivity Analysis (−25% lower cost of facility HD)	Sensitivity Analysis (+25% higher cost of facility HD)
CAPD	In-center HD	2.94	6.41	1.91
CCPD	In-center HD	4.76	37.74	2.54
Full-assisted CCPD (daily)	In-center HD	In-center HD always less costly	In-center HD always less costly	0.30
Partial-assisted CCPD (daily)	In-center HD	In-center HD always less costly	In-center HD always less costly	2.69
Full-assisted home HD (3×/wk)	In-center HD	0.09	0.65	0.05
Partial-assisted home HD (3×/wk)	In-center HD	7.12	27.43	4.09
Assisted home HD complete care (3×/wk)	In-center HD	0.02	In-center HD always more costly	In-center HD always more costly
Home HD conventional (3×/wk)	In-center HD	9.44	20.85	6.10

Abbreviations: CAPD, continuous ambulatory peritoneal dialysis; CCPD, continuous cycling peritoneal dialysis; HD, hemodialysis.

Sensitivity analysis considering a 25% reduction in the cost of in-center HD increased months required to achieve cost savings for self-care CAPD and CCPD to 6.41 and 37.74 months, respectively. Partial- and full-assist CCPD modalities were more costly at all points in this scenario. Under similar circumstances, the months required to achieve cost neutrality between self-care, full-assist, and partial-assist home HD versus in-center HD increased to 20.85, 0.65, and 27.43 months, respectively. Complete care assisted HD did not achieve cost-neutrality at any point when in-center costs were reduced by 25%. In contrast, when in-center HD costs were increased by 25%, all modalities achieved cost savings at varying time points.

With respect to patient graduation from full- and partial-assisted PD to self-care PD, this model estimates a cost savings of $36,387 and $19,632 per patient per year, respectively. For CCPD patients, this translates to an annual cost savings of $19,283 per patient when compared with in-center HD and $31,150 per patient for CAPD patients (excluding training costs). Patients graduating from assisted home HD to self-care conventional home HD can reduce annual per-patient costs by $31,201 in comparison to in-center HD (excluding training costs).

Scenario analysis results considering an increase in home HD schedules are presented in Table S1. When increasing care from 3 to 3.5 times per week, annual maintenance costs associated with conventional home HD increased by $1,809.19 per patient. Considering partial-assisted home HD, annual per-patient maintenance costs increased by $4,411.97. Full-assisted home HD and complete care assisted home HD maintenance costs both increased by $3,876.58 per patient per year under this scenario. Additionally, when using health care aides rather than licensed practical nurses, all assisted dialysis modalities become less expensive on a per-year basis in comparison to care in-center (Table S2).

## Discussion

Using a cost-minimization approach, we have established a model that includes all direct maintenance and training costs associated with assisted home dialysis modalities from a health care payer’s perspective. This model estimates that assisted thrice-weekly home HD, regardless of scenario, offers cost savings relative to in-center HD. Nevertheless, under the complete-care scenario, the difference between annual maintenance costs compared with in-center HD is the least distinct. Moreover, full- and complete care assisted home HD scenarios are less costly than self-care home HD in the short term (~10 months) due to differences in initial patient training costs. As such, they are optimal treatment options from a cost-savings perspective for patients expected to require dialysis for a limited time who are unable to perform full self-care.

Whereas this model estimates the costs associated with full-assist PD to exceed those of in-center HD, other publicly funded jurisdictions have reported similar costs between the 2 modalities.^14^^,^^30^ Notably, this model omits patient-borne costs associated with travel to and from in-center HD treatment. Transportation costs have been estimated to be ~10% of total per-patient costs for in-center patients in the United-Kingdom.^31^ As a result, in-center HD costs may be underestimated in this analysis, improving the relative attractiveness of competing modalities.

Economic gains may be realized from patients graduating from assisted modalities to full self-care. Although specific rates of graduation from assisted to full self-care are unknown in this population, research from 1 large Canadian assisted-dialysis study found graduation rates to full self-care or family-assisted care of 38%, with a median modality switch time of 31 days.^19^ It has been suggested that relaxed eligibility criteria for assisted PD and having registered nurses trained in dialysis visiting more than once per day may have positively contributed to these graduation rates.^19^ Educating and providing support for patients who are willing or able to perform self-care PD may contribute to increasing graduation rates. Education has been shown to positively influence patient modality choice and can motivate patients who are able to perform self-care to opt for such therapies.^32^

Conversely, economic disincentives such as utility costs may decrease the attractiveness of home-based modalities for patients in programs in which such costs are not covered. Although these costs are relatively minimal compared with overall costs ($404 per annum for water and $78 per annum for electricity), they may deter patients from home modalities in view of the inverse relationship between income and incidence of ESKD.^33^ As such, programs may benefit from covering such costs to incentivize home-based care.

Although assisted modalities become less attractive economically as the frequency of treatment increases, we have shown that costs per patient can be reduced when staffing models are adjusted to use less costly labor. Evidence from a Canadian pilot project demonstrated that safe and adequate care can be delivered when using health care aide equivalents to perform assisted dialysis.^34^ Thus, dialysis programs may consider adopting such a model to provide safe care in-home with reduced costs.

Assisted home modalities have been shown to offer similar outcomes to dialysis delivered in-center with respect to all-cause hospitalizations, all-cause infections, and mortality rates.^19^^,^^35^ Hospital days from dialysis-related peritonitis were found to be 3-fold higher than those from catheter-related bacteremia among patients receiving in-center care after adjustment for case-mix.^19^ Notwithstanding, evidence suggests that the increased risk for hospital days from dialysis-related infection among assisted home PD patients may disappear when undifferentiated home care nurses are supervised by PD-specific nurses, suggesting the importance of incorporating adequately trained staff in the delivery of home dialysis.^36^ We have provided costing here for assisted home HD on a typical schedule of that of an in-center patient at 3 times per week. There are likely many benefits to more frequent dialysis schedules for both in-center and assisted home HD patients.^37^^,^^38^

There are other potential cost synergies that may be possible with the expansion of assisted home dialysis programs. Increased capacity to support respite care for patients who normally perform full self-care PD may be beneficial because it has shown to be cost minimizing for failing self-care patients.^20^ Synergies with existing home-care programs can create opportunities for increased home-based care. Nurses employed in the community could perform services such as wound care in home, reducing the need for in-center visits. Economies of scale with respect to operational and administrative efficiencies may be achieved as the program grows in size, positively influencing assisted PD outcomes.^39^ Providing assisted dialysis services in nursing home settings may benefit elderly patients who are unable to perform full self-care and must otherwise travel to receive care in-center. Although research to date has demonstrated adequate clinical outcomes in this setting, further investigation with respect to quality-of-life and economic considerations is needed.^24^^,^^40^

Dialysis care in rural and remote areas can be costly in comparison to care in an urban setting.^41^ Patients may not reside in close proximity to rural dialysis centers, forcing relocation or long-distance travel to receive care, which can negatively affect quality of life.^42^ Developing an assisted-care program in such settings may enable patients who are unable to perform full self-care to remain in their communities, contributing to improved quality of life with a potential for cost savings.^26^^,^^41^ Moreover, the development of assisted-dialysis programs in rural and remote areas has been extended to satellite dialysis units as a cost-effective alternative to in-center care, allowing patients to perform self-care in a clean environment with the potential for nurse assistance.^43^ As these services expand, there is an increased opportunity for additional capacity in satellite units to become available. As such, patients’ need for relocation and transition time back to their home community may be reduced.^43^ This may also address issues related to poor housing conditions in northern communities in which potable water and lack of space are barriers to home-dialysis uptake.^44^ Additionally, as direct nurse assistance is available for patients performing self-care in satellite units, such methods of care may be suitable cost-effective alternatives for patients seeking to graduate from assisted home-based modalities who may be apprehensive of performing complete self-care.^43^ Notwithstanding, appropriate infrastructure is required, which may entail additional capital investments. Importantly, the evidence used to draw these conclusions was derived from observational studies due to the difficulty conducting randomized controlled trials wherein the setting of treatment is randomized between a hospital and home setting. As such, these findings should be interpreted with caution.

Our model has many strengths. First, this analysis uses a previously validated costing model based on actual program expenditures by the British Columbia Renal Agency to estimate direct human resources.^45^ This increases the generalizability of the model in other publicly funded jurisdictions. Second, separating maintenance and training costs allows for distinctions with respect to costs associated with the first year of care and each subsequent year. Last, this model is flexible with respect to changes in labor and costing inputs, allowing for re-estimation as needed to better reflect the cost of dialysis.

Our study has several important limitations. First, we do not account for costs taken from the societal perspective, including all home utilities, transportation, patients’ opportunity costs, or caregiver burden. Second, the results of the study reflect the costs experienced by only a single center, and heterogeneity between programs and the relative size of home dialysis programs may have a substantial influence on cost-related outcomes. As such, findings should be adapted to local circumstances and patient mix when necessary. Last, costs related to PD failure and modality switching, such as hospitalization and surgery, are not factored into calculations of cost neutrality. Although this may result in increased costs over simply initiating and maintaining in-center HD, previous research has demonstrated that patients who transition from PD to HD experience costs that are similar and not in excess to those experienced by patients who receive in-center HD as a sole modality.^8^

Dialysis therapy is a resource-intensive and costly treatment for patients with kidney failure. Assisted home-based modalities can offer similar or reduced costs in comparison to in-center HD and should be considered, particularly in programs with a high proportion of individuals relocating for in-center care. Further research is needed to determine graduation rates to full self-care, as well as costs associated with respite care, with more detailed models evaluating the impact on the entire trajectory of patient treatment with dialysis.
