# Supplementary material for: Costs of Assisted Home Dialysis: A Single-Payer Canadian Model From Manitoba
Source: Kidney Med. 2021 Jul 7;3(6):942–950.e1. doi: 10.1016/j.xkme.2021.04.019 (PMC8664694; doi:10.1016/j.xkme.2021.04.019)
Supplement: Supplementary File (PDF) — Tables S1-S2. [file mmc1.pdf]

Table S1: Scenario analysis – Annual per patient cost of dialysis maintenance therapy by modality in Manitoba, Canada (2019 Canadian dollars) - AHHD care frequency 3.5 times per week

	HHD Conventional (3.5x/wk)	Full AHHD (3.5x/wk)	Partial AHHD (3.5x/wk)	AHHD complete care (3.5x/wk)
**Human Resources (Direct)**				
Registered Nurse	$1,109.94	$1,566.45	$1,566.45	$1,566.45
Unit Clerk	$268.53	$268.53	$268.53	$268.53
LPN	$0.00	$6,145.89	$6,145.89	$6,145.89
Renal Dietician	$567.19	$567.19	$567.19	$567.19
Dialysis Technician	$1,943.01	$1,943.01	$1,943.01	$1,943.01
Clinical Pharmacist	$870.19	$870.19	$870.19	$870.19
Social Worker	$383.18	$383.18	$383.18	$383.18
HCA	$0.00	$2,677.56	$0.00	$14,321.48
**Total Human Resources**	**$5,142.03**	**$14,421.98**	**$11,744.43**	**$26,065.91**
Benefits	$1,036.12	$2,906.03	$2,366.50	$5,252.28
Vacation and Relief	$1,008.87	$2,829.59	$2,304.26	$5,114.13
Milage, Communications, etc.	$0.00	$368.63	$368.63	$368.63
Supplies - Medical, Surgical, and Laboratory	$12,132.71	$12,132.71	$12,132.71	$12,132.71
Supplies - Other (e.g. Housekeeping, Maintenance)	$2,907.54	$3,392.13	$3,392.13	$3,392.13
Drug Expenses	$3,184.98	$3,184.98	$3,184.98	$3,184.98
Equipment Expenses	$4,528.04	$4,528.04	$4,528.04	$4,528.04
Departmental Sundry / Miscellaneous	$123.99	$146.71	$141.39	$169.86
Hospital Utilities/Overhead (Electricity/Heat)	$77.76	$77.76	$77.76	$77.76
Water	$470.95	$470.95	$470.95	$470.95
Capital Cost	$3,212.65	$3,212.65	$3,212.65	$3,212.65
In-Centre Runs	$4,601.92	$4,601.92	$4,601.92	$4,601.92
**Total**	**$38,427.56**	**$52,274.09**	**$48,526.34**	**$68,571.96**

CAPD, continuous ambulatory peritoneal dialysis, CCPD, continuous cycling peritoneal dialysis, aHHD, assisted home hemodialysis, aCCPD, continuous cycling assisted peritoneal dialysis, LPN, licensed practical nurse, HCA, health care aide

Table S2: Scenario analysis – Annual per patient cost of dialysis maintenance therapy by modality in Manitoba, Canada (2019 Canadian dollars) - health care aide staffing model

	In-Centre Hemodialysis	PD (CAPD)	PD (CCPD)	HHD Conventional (3x/wk)	Full AHHD (3x/wk)	Partial AHHD (3x/wk)	AHHD complete care (3x/wk)	Partial ACCPD (daily)	Full ACCPD (daily)
**Human Resources (Direct)**									
Registered Nurse	$19,594.00	$1,933.44	$1,933.44	$1,109.94	$0.00	$0.00	$0.00	$0.00	$0.00
Unit Clerk	$1,163.65	$295.39	$295.39	$268.53	$268.53	$268.53	$268.53	$295.39	$295.39
LPN	$9,608.66	$0.00	$0.00	$0.00	$0.00	$0.00	$0.00	$0.00	$0.00
Renal Dietician	$685.35	$472.65	$472.65	$567.19	$567.19	$567.19	$567.19	$472.65	$472.65
Dialysis Technician	$645.17	$0.00	$0.00	$1,943.01	$1,943.01	$1,943.01	$1,943.01	$0.00	$0.00
Clinical Pharmacist	$428.79	$327.90	$327.90	$870.19	$870.19	$870.19	$870.19	$327.90	$327.90
Social Worker	$469.20	$383.18	$383.18	$383.18	$383.18	$383.18	$383.18	$383.18	$383.18
HCA	$0.00	$0.00	$0.00	$0.00	$5,355.11	$3,060.06	$17,338.65	$7,040.05	$12,320.10
**Total Human Resources**	**$32,594.81**	**$3,412.56**	**$3,412.56**	**$5,142.03**	**$9,387.20**	**$7,092.15**	**$21,370.74**	**$8,519.17**	**$13,799.21**
Benefits	$6,567.85	$687.63	$687.63	$1,036.12	$1,891.52	$1,429.07	$4,306.20	$1,716.61	$2,780.54
Vacation and Relief	$6,395.10	$669.54	$669.54	$1,008.87	$1,841.77	$1,391.48	$4,192.94	$1,671.46	$2,707.41
Milage, Communications, etc.	$0.00	$0.00	$0.00	$0.00	$102.21	$102.21	$102.21	$745.24	$1,304.16
Supplies - Medical, Surgical, and Laboratory	$7,844.36	$24,487.64	$36,295.34	$10,399.47	$10,399.47	$10,399.47	$10,399.47	$36,295.34	$36,295.34
Supplies - Other (e.g. Housekeeping, Maintenance)	$837.82	$333.64	$333.64	$2,907.54	$2,907.54	$2,907.54	$2,907.54	$333.64	$333.64
Drug Expenses	$6,004.55	$3,236.88	$3,236.88	$3,184.98	$3,184.98	$3,184.98	$3,184.98	$3,236.88	$3,236.88
Equipment Expenses	$580.35	$0.00	$0.00	$4,528.04	$4,528.04	$4,528.04	$4,528.04	$0.00	$0.00
Departmental Sundry / Miscellaneous	$141.15	$147.08	$206.12	$115.33	$124.28	$119.71	$148.11	$220.00	$233.29

Hospital Utilities/Overhead (Electricity/Heat)	$222.40	$77.76	$77.76	$77.76	$77.76	$77.76	$77.76	$77.76	$77.76
Water	$428.17	$0.00	$0.00	$403.67	$403.67	$403.67	$403.67	$0.00	$0.00
Capital Cost	$5,798.99	$3,212.65	$3,212.65	$3,212.65	$3,212.65	$3,212.65	$3,212.65	$3,212.65	$3,212.65
In-Centre Runs	$0.00	$0.00	$0.00	$4,601.92	$4,601.92	$4,601.92	$4,601.92	$0.00	$0.00
**Total**	**$67,415.55**	**$36,265.39**	**$48,132.13**	**$36,618.37**	**$42,663.01**	**$39,450.66**	**$59,436.23**	**$56,028.76**	**$63,980.89**

CAPD, continuous ambulatory peritoneal dialysis, CCPD, continuous cycling peritoneal dialysis, aHHD, assisted home hemodialysis, aCCPD, continuous cycling assisted peritoneal dialysis, LPN, licensed practical nurse, HCA, health care aide
